# Supplementary material for: Plant-Derived Nanovesicles from Soaked Rice Water: A Novel and Sustainable Platform for the Delivery of Natural Anti-Oxidant γ-Oryzanol
Source: Antioxidants (Basel). 2025 Jun 12;14(6):717. doi: 10.3390/antiox14060717 (PMC12189669; doi:10.3390/antiox14060717)
Supplement: Supplementary file 1 [file antioxidants-14-00717-s001.zip › antioxidants-3671234-supplementary.pdf]

A

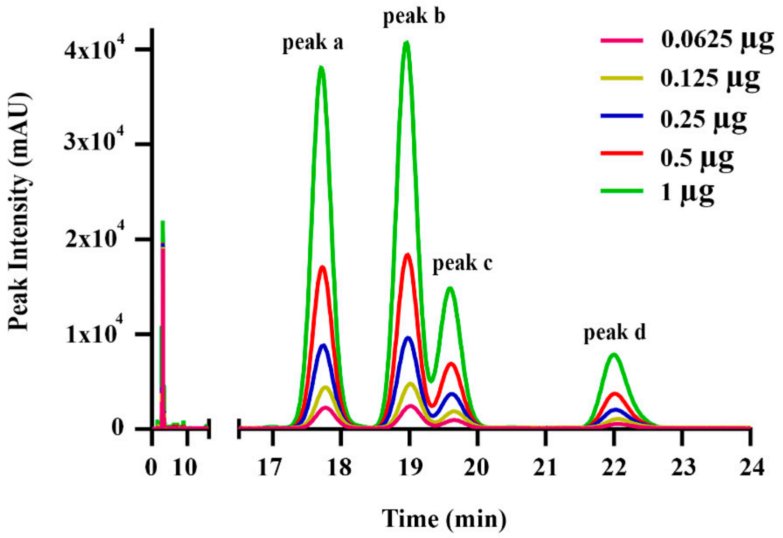

B

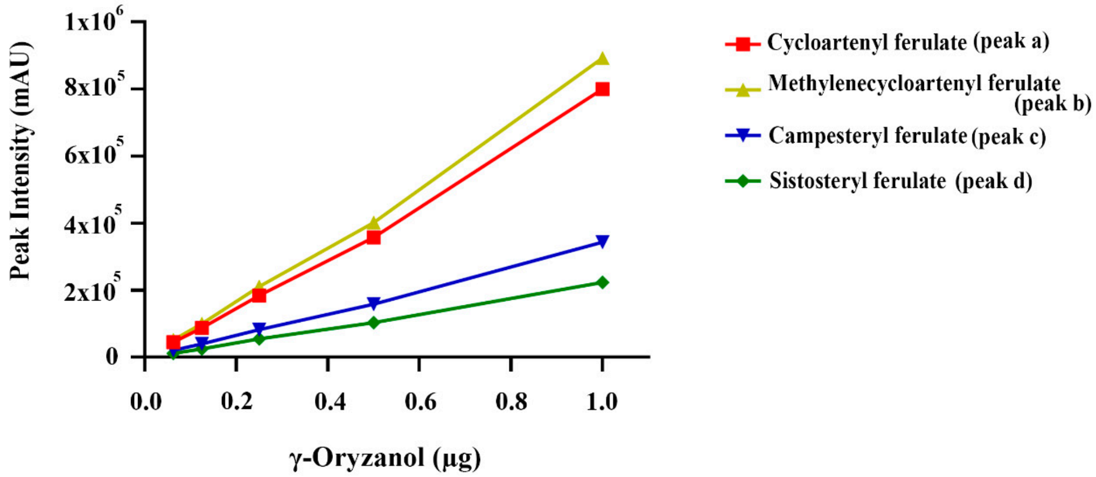

Figure S1: HPLC analysis of standard GO. A) HPLC chromatograms of increasing concentration of standard GO (0.0625, 0.125, 0.25, 0.5, 1 µg); the GO isomers peaks are indicated as peak a – Cycloartenyl ferulate, peak b – Methylenecycloartanyl ferulate, peak c – Campesteryl ferulate, peak d – Sistosteryl ferulate. B) Calibration curve of GO isomers (peak intensity, mAU against GO concentration, µg) for the increasing concentration of standard GO.
